# Non-invasive monitoring of pH and oxygen using miniaturized electrochemical sensors

**DOI:** 10.1186/s12967-021-02923-1

**Published:** 2021-06-08

**Authors:** Mehrdad Farrokhi, Seyedeh Parisa Manavi, Fatemeh Taheri

**Affiliations:** 1 Eris Research Institute, Tehran, Iran; 2 School of Medicine, Isfahan University of Medical Sciences, Isfahan, Iran; 3 Faculty of Pharmacy and Pharmaceutical Sciences, Tehran Medical Sciences, Islamic Azad University, Tehran, Iran

Dear Editor,

We read with great interest a recent published article by Pla et al. [1] in Journal of Translational Medicine. In this study, the authors investigated some variables including partial pressure of oxygen, pH, lactate, bicarbonate, and potassium in different time-points of hypoxia induction (i.e., basal, hypoxia-acidosis, and recovery periods). Therefore, they compared these variables between different time-points of measurement in one sample of 27 New Zealand White male rabbits. Since their comparisons were within-group, they were dependent to each others. However, they stated in abstract and statistical analysis section of the methods that one-way analysis of variance (ANOVA) was used to compare variables between different time-points of measurement. One-way ANOVA is used to compare numerical variables with normal distribution between more than two independent groups [2–6]. Because the comparisons in this study was performed between different time-points of measurements in one group of animals, the authors must assess normal distribution of numerical variables and then use repeated measures ANOVA or Friedman test [7–10]. Furthermore, they used student’s *t*-test for comparison of numerical variables between each two time-points of measurement, while they must use paired *t*-test or Wilcoxon for comparison of numerical variables between two time-points of measurement.

## Data Availability

Not applicable.
